# Diverse mechanisms of taste coding in *Drosophila*

**DOI:** 10.1126/sciadv.adj7032

**Published:** 2023-11-17

**Authors:** Hany K. M. Dweck, John R. Carlson

**Affiliations:** ^1^ Department of Molecular, Cellular and Developmental Biology, Yale University, New Haven, CT 06511, USA.; ^2^ Department of Entomology, The Connecticut Agricultural Experiment Station, New Haven, CT 06511, USA.

## Abstract

Taste systems encode chemical cues that drive vital behaviors. We have elucidated noncanonical features of taste coding using an unconventional kind of electrophysiological analysis. We find that taste neurons of *Drosophila* are much more sensitive than previously thought. They have a low spontaneous firing frequency that depends on taste receptors. Taste neurons have a dual function as olfactory neurons: They are activated by most tested odorants, including *N*,*N*-diethyl-*meta*-toluamide (DEET), at a distance. DEET can also inhibit certain taste neurons, revealing that there are two modes of taste response: activation and inhibition. We characterize electrophysiological OFF responses and find that the tastants that elicit them are related in structure. OFF responses link tastant identity to behavior: the magnitude of the OFF response elicited by a tastant correlated with the egg laying behavior it elicited. In summary, the sensitivity and coding capacity of the taste system are much greater than previously known.

## Teaser

Non-canonical features of taste coding are elucidated with an unconventional kind of electrophysiology.

## INTRODUCTION

Taste coding is unexpectedly intricate and poorly understood. The primary sensory cells are diverse, and they express not one but a multiplicity of receptors that may interact in complex ways (*1*–*12*). These receptors collectively transduce an enormous variety of molecular cues that carry critical information about an animal’s environment (*3*, *7*, *8*). Sensitive detection and interpretation of this information underlie vital decisions such as whether to feed on a potential food source (*3*, *7*, *8*).

The main taste organ of the *Drosophila* head, the labellum, provides an excellent system in which to address fundamental principles of taste coding. It is numerically simple, anatomically stereotyped, and amenable to convenient electrophysiological analysis (*2*, *5*, *6*). It also drives a variety of behaviors that range over time scales of seconds to hours (*5*, *6*, *13*, *14*).

**Fig. 1.**
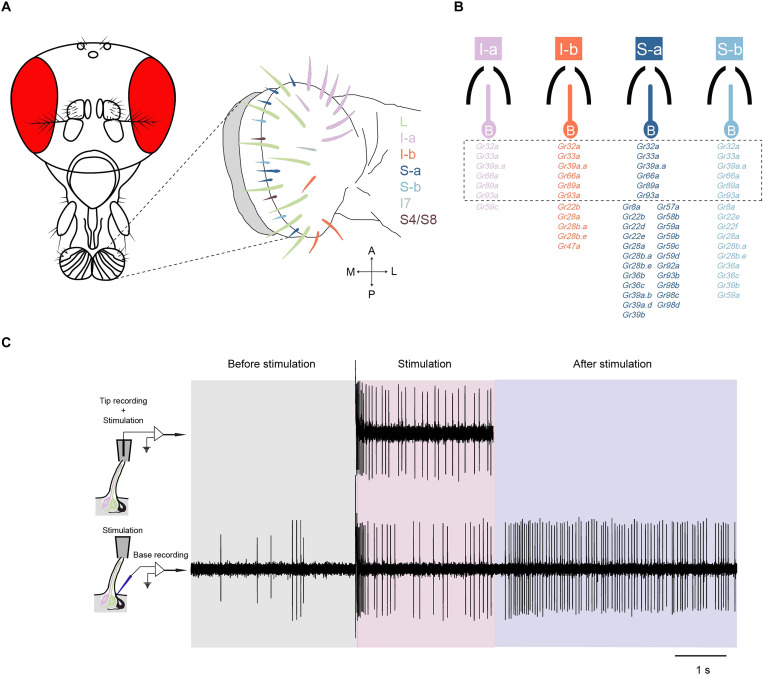
Labellar sensilla and taste electrophysiology techniques. (**A**) Left: Fly head. Right: Map of taste sensilla on the labellum, showing L, I, and S sensillum classes. (**B**) The four classes of sensilla that contain a bitter-sensitive neuron, indicated by “B.” These sensilla all contain other neurons that are not shown. The bitter-sensitive neuron of each sensillum class expresses a different subset of *Gr* genes, as indicated. Six of these genes, indicated by dashed lines, are expressed in all four classes. (**C**) Taste electrophysiology techniques. Top: Tip recording. Spikes are observed only during period when the tastant [1 mM denatonium benzoate (DEN)] makes contact with the sensillum. Bottom: Base recording. Spikes are observed before, during, and after contact. I-a sensillum. An OFF response is visible after the stimulus is removed.

The labellum contains 31 taste sensilla, each with a pore at the tip through which tastants can enter. These sensilla fall into classes based on size: large (L), intermediate (I), and small (S) (*15*–*17*). These classes can, in turn, be divided into subclasses, e.g., I-a, I-b, S-a, and S-b, based on response profile and expression of *Gustatory receptor* (*Gr*) genes (Fig. 1, A and B) (*5*). Each sensillum is innervated by up to four taste neurons (*5*, *16*). One neuron is sensitive to bitter compounds and generates an action potential with a large amplitude (*5*, *6*, *18*–*23*) ; another is sensitive to sugars and generates a smaller spike in most sensillum types (*23*–*25*). Many sensilla contain a neuron sensitive to water or low osmolarity (*26*, *27*), and most taste sensilla have a mechanosensory neuron at their base (*7*, *16*, *28*–*31*).

For the past 50 years, virtually all electrophysiological analysis of *Drosophila* taste has used a method called tip recording, in which a glass electrode is placed over the tip of a taste sensillum (*22*, *32*). The electrode delivers a tastant to the sensillum and simultaneously records the neuronal responses. This method thus measures the responses of taste neurons only during contact with the stimulus (Fig. 1C, top) (*2*, *5*, *6*). Accordingly, neural activity before or after stimulus contact is not measured.

To overcome these limitations, we have analyzed taste coding using an alternative method that we call base recording (*33*, *34*). This method allows measurement of the activity of a taste neuron before, during, and after the stimulus (Fig. 1C, bottom). In addition, unlike tip recording, compounds can also be delivered as vapors. Base recording has allowed us to observe and quantify features of taste electrophysiology that had not been measured before. We have also been able to examine the evolution of these features, since base recording can be applied to other species without the need for genetic tools such as drivers and effectors. The method provides great sensitivity as well as high spatial and temporal resolution. One can distinguish the action potentials of different neurons, including the mechanosensory neuron whose activity can cause confounding movement artifacts if not properly identified.

Here, we identify and characterize features of taste coding using base recording. We measure the spontaneous activity of Gr neurons (GRNs). We are unaware of any previous measurements of spontaneous activity in the taste neurons of any organism. The spontaneous firing frequency of bitter, sugar, and water GRNs is low compared to that of olfactory receptor neurons (ORNs), a feature that has been conserved for ~65 million years. The spontaneous firing of a bitter neuron is dependent on the Grs that it expresses, suggesting a model for the mechanism underlying spontaneous activity. We find that *N*,*N*-diethyl-*meta*-toluamide (DEET), coumarin (COU), and most members of a panel of 47 odorants activate GRNs at a distance; thus, GRNs can function as ORNs. DEET can also inhibit the spontaneous activity of GRNs in certain contexts, revealing that there are two modes of GRN response: activation and inhibition. Unexpectedly, base recording reveals that bitter and sugar neurons are much more sensitive than previously thought, by more than two orders of magnitude in some cases. We characterize electrophysiological OFF responses from a broad panel of tastants and find that tastants that elicit OFF responses are related in structure. Most unexpectedly, the egg-laying behavior elicited by a tastant could be predicted from the magnitude of OFF responses it produced. In summary, the coding capacity of the taste system extends well beyond what was previously known.

## RESULTS

### Spontaneous activity of taste neurons

**Fig. 2.**
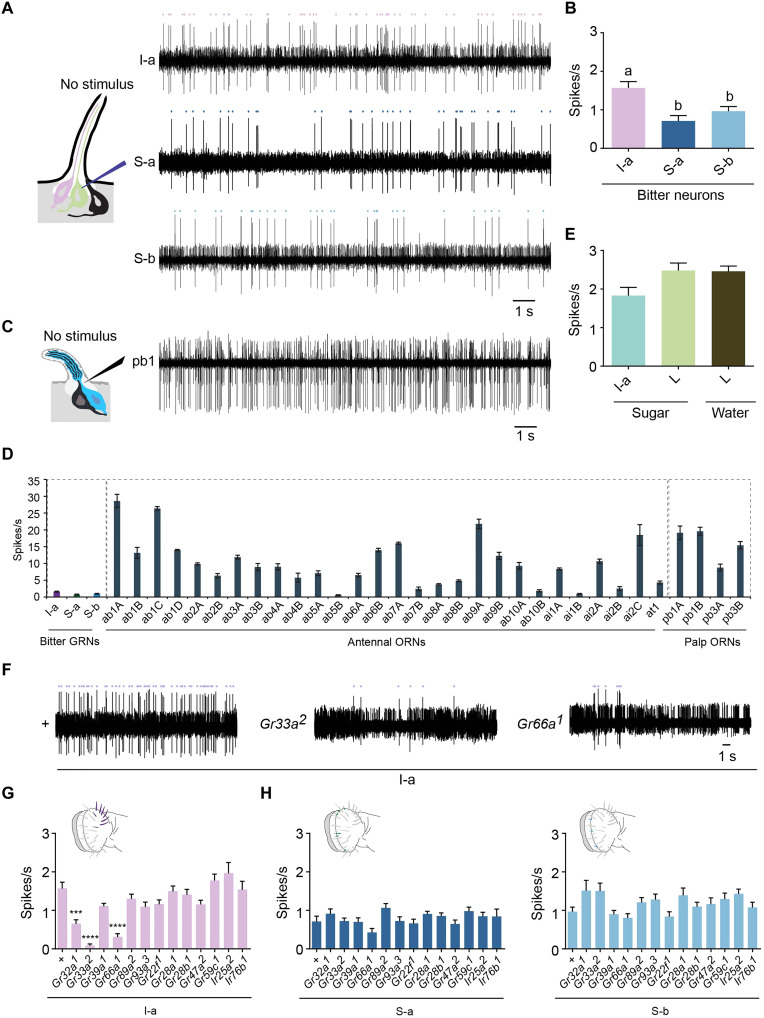
Spontaneous firing activity of bitter neurons and its mechanism. (**A**) Example traces of spontaneous activity from I-a, S-a, and S-b. Dots indicate spikes of bitter neurons. (**B**) Spontaneous activities of I-a, S-a, and S-b. Bars with “a” and “b” above are significantly different. *P* < 0.05, one-way ANOVA followed by Tukey’s multiple comparison test; *n* = 9 to 17. Error bars are SEM. (**C**) Example trace of spontaneous activity from the maxillary palp sensillum type pb1. Action potentials of two amplitudes, from two ORNs in the sensillum, are visible. (**D**) Spontaneous activities of 32 ORNs. *n* = 5. Error bars are SEM. (**E**) Spontaneous activities of sugar and water neurons. *n* = 5 to 7. Error bars are SEM. (**F**) Example traces of spontaneous activities of I-a from the *w Canton-S* control (+), *Gr33a^2^*, and *Gr66a^1^*. (**G**) Spontaneous activities of I-a from the indicated genotypes. + is *w Canton-S*. ***P < 0.001 and ****P < 0.0001, one-way ANOVA followed by Dunnett’s multiple comparison test; *n* = 7 to 17. Error bars are SEM. (**H**) Spontaneous activities of S-a and S-b from the indicated genotypes. *P* > 0.05, one-way ANOVA followed by Dunnett’s multiple comparison test; *n* = 6 to 11 for S-a and *n* = 5 to 9 for S-b. Error bars are SEM.

We recorded the spontaneous activity of three classes of bitter taste neurons, I-a, S-a, and S-b, in the female labellum. For each individual measurement, we recorded the spontaneous activity for 100 s. We found that bitter neurons have low spontaneous firing rates. The spontaneous activity of I-a bitter neurons was 1.6 ± 0.2 spikes/s, whereas those of S-a and S-b bitter neurons were even lower: 0.7 ± 0.1 and 1.0 ± 0.1 spikes/s [Fig. 2, A and B; *P* < 0.05; one-way analysis of variance (ANOVA), Tukey’s multiple comparison test; *n* = 9 to 17]. The spontaneous firing rates of these neurons were the same in starved flies (fig. S1A). The rates were also low in males, although somewhat higher than those of females in the case of I-a neurons (fig. S1A; *P* < 0.05, one-way ANOVA, Tukey’s multiple comparison test; *n* = 5 to 7).

How do the spontaneous firing rates of bitter GRNs compare with those of ORNs? In a direct comparison in the same strain of flies on the same electrophysiology rig, we analyzed the spontaneous firing rates of 32 different ORN classes by inserting an electrode into the base of olfactory sensilla and again recording for 100 s in the absence of airflow. The ORNs varied in their spontaneous firing activities, consistent with less extensive earlier reports (*35*–*37*), but the great majority of these ORNs had much higher spontaneous firing activities than those of bitter neurons (Fig. 2, C and D; *P* < 0.05 for each bitter neuron, one-way ANOVA, Dunnett’s multiple comparison test; *n* = 9 to 17 for bitter neurons and *n* = 5 for ORNs).

To determine whether a low spontaneous firing rate is a conserved feature, we measured the spontaneous firing activity of bitter neurons in five other species that occupy diverse habitats. These species diverged from *Drosophila melanogaster* at times ranging from 2 to 3 million years ago (*Drosophila simulans*) to ~63 million years ago (*Drosophila virilis*) (*38*–*42*). Each species contains a population of sensilla in similar positions to those of I-a sensilla in *D. melanogaster* (fig. S1B). These sensilla all contain a neuron that produces large spikes and that responds to bitter compounds. In all species, the spontaneous firing activity of this bitter neuron was <2 spikes/s (fig. S1, C and D). In *Drosophila erecta*, *Drosophila mojavensis*, and *D. virilis*, the spontaneous firing activity was lower than that in *D. melanogaster* and *Drosophila sechellia* (*P* < 0.05, one-way ANOVA, Tukey’s multiple comparison test; *n* = 5 to 12) (fig. S1, C and D). The simplest interpretation of these results is that the relatively low firing rate of I-a bitter neurons is a conserved feature.

Measuring the spontaneous rate of the other taste neuron in I-a sensilla, which responds to sugar, is challenging: Under unstimulated conditions, its amplitude is difficult to distinguish from that of the mechanosensory neuron. However, in a small fraction of cases (<5%), it was possible to identify the spikes of the sugar neuron clearly, and their spontaneous firing frequencies were also <2 spikes/s (Fig. 2E and fig. S1E). We also examined L sensilla, and, in the small fraction of cases in which we could clearly identify sugar- and water-sensitive neurons in the absence of stimulation, their spontaneous firing frequencies were <3 spikes/s (Fig. 2E and fig. S1E). In summary, the spontaneous firing frequencies of these bitter, sugar and water neurons are very low, and different GRNs exhibit different spontaneous frequencies.

### Mechanism of spontaneous activity

Our new ability to measure spontaneous firing allowed us to examine its mechanism. Accordingly, we tested the hypothesis that the spontaneous firing of a bitter neuron depends on the receptors that it expresses. We measured the spontaneous activities of I-a, S-a, and S-b bitter neurons in a series of receptor mutants, including six mutants of *Gr* genes that are expressed in all bitter neurons of the labellum (*Gr32a*, *Gr33a*, *Gr39a*, *Gr66a*, *Gr89a*, and *Gr93a*) and five mutants of *Gr* genes that are expressed in some bitter neurons (*Gr22f*, *Gr28a*, *Gr28b*, *Gr47a*, and *Gr59c*) (Fig. 1B). *Gr32a*, *Gr33a*, *Gr39a*, *Gr66a*, and *Gr89a* were generated via CRISPR-Cas9 genome editing and back-crossed to the control stock for five generations to minimize genetic background effects.

The spontaneous activity of I-a bitter neurons was reduced by mutations in three *Gr* genes (*Gr32a*, *Gr33a*, and *Gr66a*) (*P* < 0.05, one-way ANOVA followed by Dunnett’s multiple comparison test; *n* = 7 to 17), with *Gr33a* and *Gr66a* having particularly severe effects (Fig. 2, F and G). No mutations had a significant effect on the spontaneous activities of S-a and S-a bitter neurons. We note that the number of receptors expressed in these three neuronal classes varies widely: I-a bitter neurons express 7 Grs, while those in S-a express 29 Grs and those in S-b express 16 Grs (*5*, *6*). Thus it is possible that the neuron in which we identified phenotypes, the I-a neuron, has the least genetic redundancy.

### Coding of bitter tastants without taste

Having first examined the activity of bitter neurons before stimulation, we next sought to examine their activity during stimulation. We selected a panel of structurally diverse bitter compounds, including naturally occurring alkaloids, terpenoids, and phenolic compounds. These compounds are aristolochic acid (ARI), berberine (BER), caffeine (CAF), COU, DEET, denatonium benzoate (DEN), escin (ESC), lobeline (LOB), quinine (QUI), saponin (SAP), d-(+)-sucrose octaacetate (SOA), sparteine (SPS), strychnine (STR), theophylline (TPH), and umbelliferone (UMB).

**Fig. 3.**
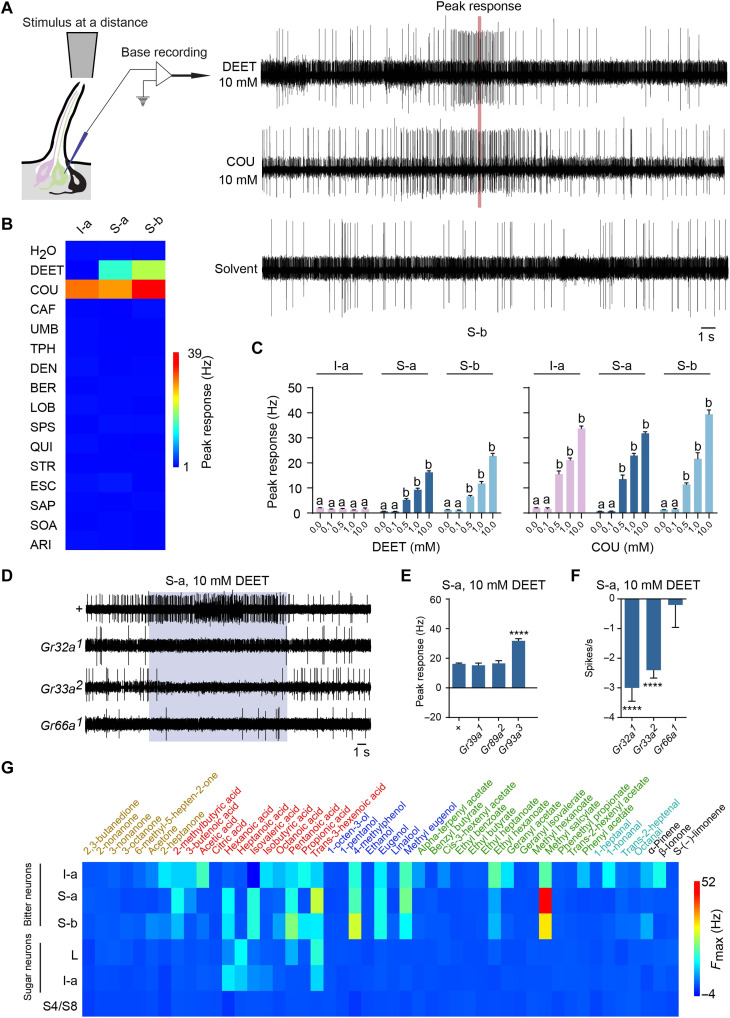
Coding of tastants without taste. (**A**) Example traces of responses of S-b to the vapors of 10 mM DEET, 10 mM COU, and the solvent control, water. The red line indicates the peak response. The stimulus was moving toward the sensillum before the peak response and away from the sensillum after the peak response. (**B**) Heatmaps of responses from I-a, S-a, and S-b to the solvent (water) and a battery of 15 chemical compounds. *n* = 5 to 6. (**C**) Responses to the vapors of a range of concentrations of DEET and COU. *n* = 5. Error bars are SEM. Bars indicated with a are different from bars indicated with b. One-way ANOVA followed by Tukey’s multiple comparison test. (**D**) Representative traces of responses from S-a of control (*+*), *Gr32a^1^*, *Gr33a^2^*, and *Gr66a^1^* to the vapor of 10 mM DEET. The spikes of lower amplitude during the middle of the stimulus period in the control trace are from the mechanosensory neuron. (**E**) Excitation of S-a by 10 mM DEET in the indicated genotypes. ****P < 0.0001, one-way ANOVA followed by Dunnett’s multiple comparison test; *n* = 5. Error bars are SEM. (**F**) Inhibition of S-a by 10 mM DEET in the indicated genotypes. ****P < 0.0001, Mann-Whitney test; *n* = 5. Error bars are SEM. (**G**) Heatmaps of responses from I-a, S-a, and S-b to a battery of 47 volatile compounds at a 10% concentration. *n* = 5.

Taste is defined as a sensation that occurs upon contact with a substance. However, when screening I-a, S-a, and S-b taste neurons with this tastant panel, we observed responses to two compounds before contact was made. As a capillary containing either DEET or COU approached the sensillum, the spike frequency of the bitter neuron began to increase, showing a robust response (Fig. 3A). After the capillary was withdrawn, the spike frequency returned to baseline. The peak frequencies we observed when using 10 mM stimuli are shown in Fig. 3B. These frequencies are dose dependent for both DEET and COU (Fig. 3C). They are also neuron dependent, in that responses to DEET were observed in S-a and S-b but not I-a (Fig. 3, B and C).

DEET is used globally as an insect repellent and acts on many insect species (*43*), but the underlying mechanism is controversial. DEET acts on olfactory neurons at a distance (*44*–*47*) and on taste neurons when in contact with them (*48*, *49*), but activation of taste neurons at a distance has not previously been reported to our knowledge and invited further investigation.

We first asked whether the response of taste neurons to the vapor of DEET was evolutionarily conserved. We found that it was conserved in S-a sensilla in all of four other species tested, *D. simulans*, *D. erecta*, *D. sechellia*, and *Drosophila suzukii.* (fig. S2, A and B).

To explore the molecular basis of the taste neuron response to DEET vapor, we examined the response of S-a neurons in a series of *Gr* mutants. Several notable results were obtained:

1) The response to DEET vapor depended on three Grs: Gr32a, Gr33a, and Gr66a (Fig. 3D). The same Grs were required for response to DEET when DEET was delivered as a conventional taste solution (*6*, *48*). Gr39a and Gr89a were not required when DEET was delivered via either method (Fig. 3E) (*6*).

2) In *Gr32a* and *Gr33a* mutants, not only was the excitatory response absent, but an inhibitory response was observed (Fig. 3, D and F). Thus, in these contexts, DEET acts as an inhibitor as opposed to an activator of GRNs.

3) In a *Gr93a* mutant, DEET produces a greater response than in control (Fig. 3E). There is ample precedent for increased taste responses in *Gr* mutant backgrounds, which have been interpreted as a loss of inhibition that occurs in wild type when one Gr binds to another (*6*). However, increased response to DEET in *Gr93a* was not observed when DEET was delivered as a conventional tastant (*6*), which could reflect a difference in the molecular underpinnings of response to DEET vapor versus DEET solution.

4) The trace for (+, S-a, 10 mM DEET) in Fig. 3D illustrates the occasional firing of the mechanosensory neuron during a recording, visible as a burst of small amplitude spikes during the middle of the DEET response. This firing is observed because of motion of the labellum while applying the taste stimulus. An advantage to electrophysiology is that this firing can be identified as a mechanosensory signal and not confounded with the taste signal.

### Taste neurons as olfactory neurons

We then wondered whether other compounds could activate taste neurons at a distance. We screened taste sensilla with a battery of 47 diverse odorants, prioritizing compounds that are found in fruits and are known to act on olfactory neurons (*35*–*37*, *50*, *51*). The panel included ketones, organic acids, alcohols, esters, aldehydes, and terpenes.

Unexpectedly, we found that the vapor of many compounds activated bitter taste neurons (Fig. 3G). I-a, S-a, and S-b bitter neurons all responded to compounds of multiple chemical classes. We examined in more detail the responses to 4-methylphenol, methyl eugenol, and methyl salicylate, which were among the compounds that elicited the strongest responses. These responses were dose dependent, with responses observed to the vapor of 0.1% concentrations in some cases (fig. S2, C and D).

Sugar neurons of both L and I-a sensilla were also activated at a distance but only by vapors of organic acids (Fig. 3G). We tested four of these acids in detail and found that the responses were also dose dependent (fig. S2, E and F). Another class of sensilla, the S4/S8 sensilla, which do not express Gr receptors and do not respond to bitter compounds (*5*), did not respond to vapors of any tested compound (Fig. 3G).

In summary, we found that taste neurons respond to the vapor of a wide variety of diverse compounds. These responses might represent an evolutionary mechanism to allow a fly to evaluate its environment at a very close distance, e.g., on a food source, when its olfactory system has adapted.

### Unexpected taste sensitivity

Having examined the activity of taste neurons in the absence of a stimulus and when a stimulus is at a distance from them, we next examined the activity of taste neurons when a tastant makes contact with the sensillum that houses them, i.e., the canonical taste mode. We began by recording the activity elicited by well-studied bitter tastants from the I-a sensillum (*2*, *5*, *6*). In contrast to previous work, which used conventional tip recording (*2*, *5*, *6*, *17*–*25*, *48*), we used base recording for this analysis. Base recording is simpler and thereby avoids some complications. First, in tip recording, tricholine citrate (TCC) is typically added to solutions of bitter compounds to suppress the response of the water neuron and to act as an electrolyte (*5*). The response to TCC alone is then often subtracted from the response to the tastant solution; the difference is interpreted as the response to the tastant per se. We have neither needed nor used TCC in our base recordings; we can easily distinguish the spikes of the bitter and water neurons (fig. S3B). A second simplification of base recording is that the stimulus is delivered in the absence of a recording electrode, which contains a metal wire, often silver. Metals elicit taste responses (*13*, *52*–*56*) and may have complicated effects on taste neuron physiology.

**Fig. 4.**
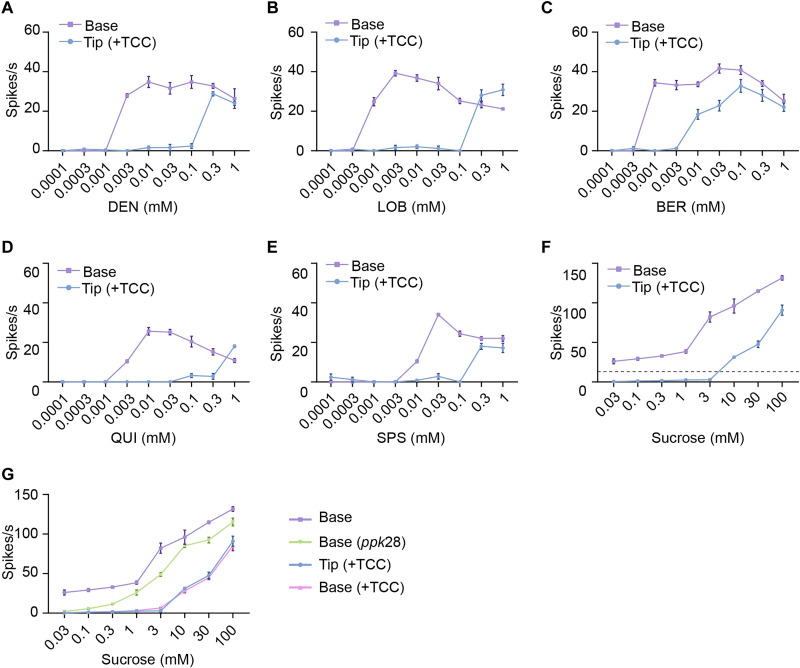
Sensitivity of taste neurons. (**A** to **E**) Responses of I-a to a range of concentrations of DEN (A), LOB (B), BER (C), QUI (D), and SPS (E) using base and tip recording techniques. *n* = 5. Error bars are SEM. Genotype is *Canton-S.* The dose-response analysis in (A) was scored blind; the experimenter did not know the concentrations. (**F**) Responses of L sensilla to a range of concentrations of sucrose using base and tip recording techniques. *n* = 5. Error bars are SEM. The dashed line indicates the mean response to water, evaluated by base recording; *n* = 5. (**G**) Responses of L sensilla to a range of concentrations of sucrose as measured with the indicated methods. Genotypes are *Canton-S* except as indicated. *n* = 5. Error bars are SEM.

We were surprised to find via base recording that bitter neurons are much more sensitive than previously thought (*2*, *5*, *6*, *18*, *19*, *21*, *48*, *49*). For example, using base recording, we observed strong responses to DEN at a 0.003 mM concentration, much lower than with tip recording (Fig. 4A). The difference in responses is even greater in the case of LOB: Strong responses are observed at a concentration that is more than two orders of magnitude lower (Fig. 4B). Likewise, threshold responses to BER, QUI, and SPS are all much lower when measured via base recording (Fig. 4, C to E). The lower sensitivity of tip recordings likely arises because either TCC or metal ions suppress the bitter responses (*52*, *57*).

Sugar responses from L sensilla were also observed at a lower threshold when measured via base recording, although these sugar responses are complicated by the similar amplitudes of sugar and water neurons; thus, the spike counts for sugar response include both the responses to sugar and to water in the base recordings and do not vanish at very low sucrose concentrations (Fig. 4F). To examine the response to sugar per se, we carried out the same dose-response analysis using base recording in a *ppk28* mutant (*26*, *27*), which abolishes water response (Fig. 4G). We found that the response curve is shifted down in *ppk28*, showing no response at very low sucrose concentrations. Why are the responses of the *ppk28* mutant greater than the responses using tip recording? When we added TCC to the stimuli used in base recordings, the responses were identical to those measured by tip recording (Fig. 4G; *P* > 0.05 at all concentrations, Mann-Whitney test). The simplest interpretation of these results is that TCC suppresses not only the water response but also some portion of the response to sucrose per se, consistent with an earlier suggestion (*58*).

In summary, taste neurons of the fly detect bitter compounds and sugar with much greater sensitivity than had been previously appreciated. These low thresholds may reflect the need to detect low concentrations of these compounds in a variety of fruits.

### OFF responses: The temporal dynamics of taste electrophysiology

**Fig. 5.**
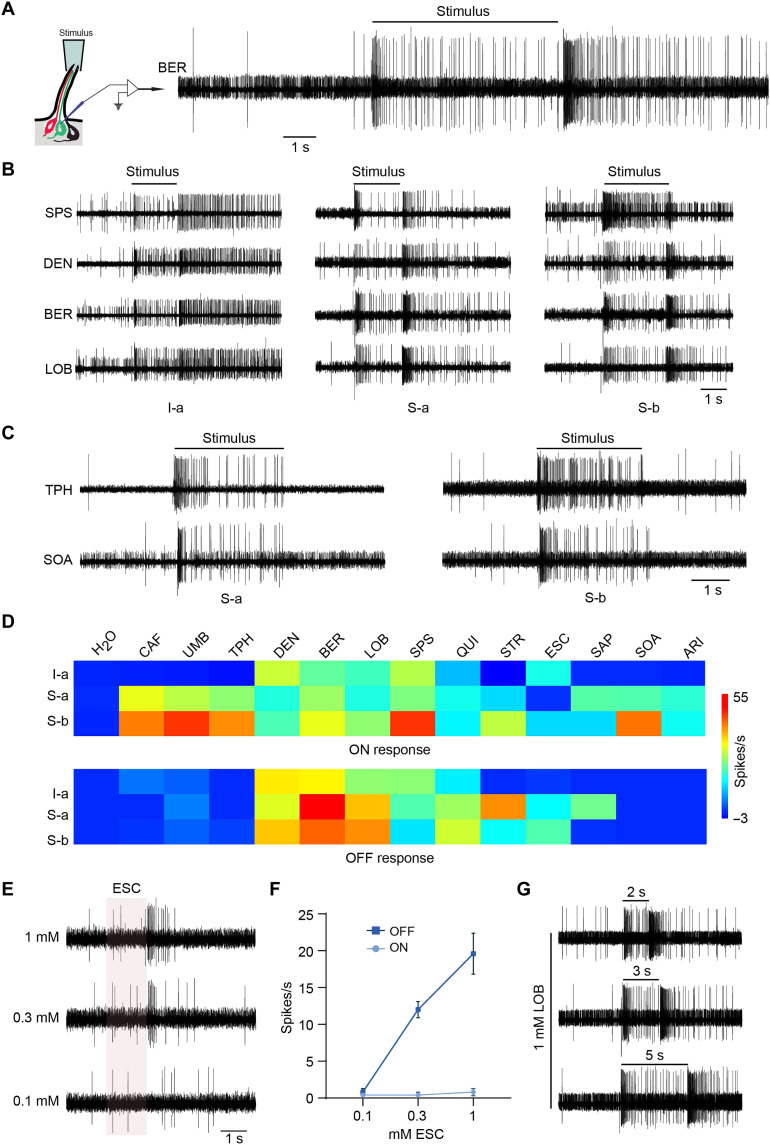
OFF responses. (**A**) Example trace of ON and OFF responses from I-a to 1 mM BER. (**B**) Example traces of ON and OFF responses from I-a, S-a, and S-b. Concentrations are indicated in Materials and Methods. (**C**) Example traces of responses from S-a and S-b to 10 mM TPH and 1 mM SOA. (**D**) Heatmaps of ON (top) and OFF (bottom) responses from I-a, S-a, and S-b to the solvent (H_2_O) and a battery of 13 bitter compounds. Concentrations are indicated in Materials and Methods. *n* = 5 to 7. (**E**) Sample traces of S-a responses to ESC. (**F**) Dose-response analysis of ON and OFF responses of S-a to ESC. *n* = 5. (**G**) Example traces of responses from S-b to 1 mM LOB with different stimulus durations.

Using base recording, we observed a notable electrophysiological response that had never been possible to observe with tip recording: OFF responses. Following stimulus onset, there is an increase in spike frequency, which subsequently declines, but then increases sharply upon stimulus offset (large spikes in Fig. 5A). This OFF response is similar to responses recently observed with three bitter tastants, QUI, DEN, and LOB, via Ca^2+^ imaging (*59*–*61*).

To assess the prevalence of this feature of taste electrophysiology, we systematically screened I-a, S-a, and S-b sensilla with a panel of 13 bitter compounds. Responses were quantified by counting the number of spikes generated over a 500-ms period after stimulus onset and offset. All of these compounds produced an increase in action potential frequency at stimulus onset, i.e., ON responses, from at least some neuron types (Fig. 5, B to D). At stimulus offset, eight of these bitter compounds, but not five others, produced strong OFF responses (Fig. 5, B to D) in at least one neuron type at the test concentrations.

OFF responses were observed in all tested classes of bitter neurons, e.g., I-a, S-a, and S-b bitter neurons (Fig. 5, B and D). In each of the three neuron classes, there are examples of bitter compounds that produce strong ON responses but not strong OFF responses at the tested concentrations: e.g., ESC in I-a; CAF in S-a; and UMB in S-b. Unexpectedly, the reciprocal was observed for ESC in S-a: we observed an OFF response but no ON response across a range of concentrations, demonstrating that an ON response is not a necessary prerequisite for an OFF response (Fig. 5, D to F).

Of the 19 cases in which both ON and OFF responses were observed, there was no correlation between their magnitudes (*r* = 0.1; *P* = 0.8, Spearman correlation). OFF responses were stronger than ON responses in 58% of these cases, whereas in only two cases (11%), OFF responses were weaker than the ON responses (SPS, S-b; STR, S-b); in 32% of the cases, the responses were indistinguishable (*P* < 0.05, Mann-Whitney test for all comparisons). The magnitude of the OFF stimulus was the same following a 2-, 3-, or 5-s stimulus (Fig. 5G and fig. S3A; *P* > 0.05, one-way ANOVA, Tukey’s multiple comparison test; *n* = 5), in agreement with earlier results (*59*). We did not observe OFF responses when sucrose, glucose, NaCl, or water were tested against a variety of L, I-a, S-a, or S-b sensilla (fig. S3B).

**Fig. 6.**
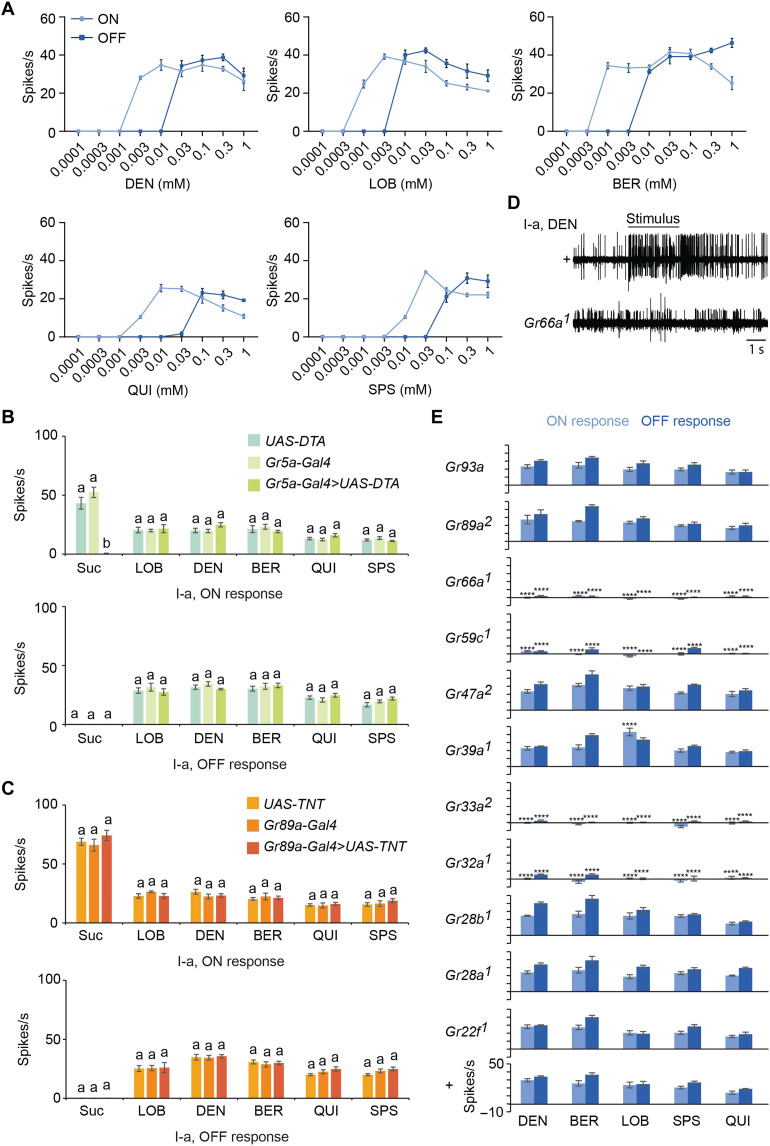
Cellular and molecular basis of ON and OFF responses to bitter compounds. (**A**) ON and OFF responses from I-a to a range of concentrations of DEN, LOB, BER, QUI, and SPS. *n* = 5. Error bars are SEM. For ease of comparison, the ON responses shown in Fig. 4 (A to F) are reproduced in (A). (**B** and **C**) Mean ON (top) and OFF (bottom) responses from I-a of the indicated genotypes to 100 mM sucrose, 1 mM LOB, 1 mM DEN, 1 mM BER, 1 mM QUI, or 10 mM SPS. *n* = 5 to 7. Error bars are SEM. Columns marked by a and b are statistically different. One-way ANOVA followed by Tukey’s multiple comparison test. (**D**) Example traces of responses from I-a of control *w Canton-S* (*+*) and *Gr66a^1^* flies to 1 mM DEN. (**E**) ON and OFF responses from I-a, in the indicated genotypes. +, *w Canton-S*. ****P < 0.0001, one-way ANOVA followed by Dunnett’s multiple comparison test; *n* = 5 to 9. Error bars are SEM.

The threshold for the OFF responses to DEN in I-a sensilla was between 0.01 and 0.03 mM; the thresholds for LOB and BER were lower, i.e., between 0.003 and 0.01 mM. Thresholds for QUI and SPS were higher but below 0.01 mM (Fig. 6A). All of these thresholds were higher, however, than for the corresponding ON responses.

### The cellular and molecular basis of the OFF responses

We next examined the cellular and molecular basis of the OFF responses. We focused on the I-a bitter neuron, which has only a single neighboring taste neuron, a sugar neuron, in its sensillum (*5*). We found that killing the sugar neuron using *UAS-DTA* (diphtheria toxin) did not affect the OFF response to any of five bitter compounds (*P* > 0.05, one-way ANOVA, Tukey’s multiple comparison test; *n* = 5) (Fig. 6B).

We then asked whether the OFF response depends on the activity of neurons postsynaptic to the bitter neuron, by blocking synaptic transmission from bitter neurons with *UAS-TNT* (tetanus toxin). Again, the spike frequency of the OFF response was unaffected (*P* > 0.05; *n* = 5) (Fig. 6C), confirming and extending earlier results found when synaptic transmission was blocked with *shibire^ts^* (*59*).

Next, we examined the OFF responses of I-a bitter neurons in 11 *Gr* mutants, using all five of the bitter compounds that produced OFF responses in these neurons. OFF responses were essentially eliminated by four *Gr* mutations, *Gr32a*, *Gr33a*, *Gr59c*, and *Gr66a*, all of which eliminated ON responses as well. Mutations of seven other *Gr* genes, including three expressed in the I-a bitter neuron, reduced neither the OFF nor ON response. Our results support the conclusion that these OFF responses depend on the same receptors as ON responses (*59*).

### Evolutionary shifts in OFF responses

We wondered whether OFF responses were conserved in evolution. An advantage of electrophysiology is that it can be used to investigate a wide variety of fly species easily, without the difficulty of introducing transgenes into them. Accordingly, we analyzed I-a bitter neurons in *D. simulans*, *D. sechellia*, *D. erecta*, *D. mojavensis*, and *D. virilis* with five bitter compounds that produced ON and OFF responses in *D. melanogaster*.

All species showed OFF responses, but there are major evolutionary shifts in the response patterns (fig. S4). For example, in the case of BER, in *D. mojavensis*, both ON and OFF responses are smaller than in *D. melanogaster;* by contrast, in *D. virilis*, they are both larger (fig. S4, A and C; *P* < 0.05, one-way ANOVA, Dunnett’s multiple comparison test; *n* = 5 for ORNs). These different responses may represent evolutionary adaptations that serve the ecological needs of these species.

In *D. mojavensis*, there is no OFF response to SPS; the ON response is somewhat smaller than in *D. melanogaster* but is consistently observed (fig. S4, B and C). Thus, ON and OFF responses can be uncoupled over evolutionary time. The response to SPS has become similar to the response of S-b in *D. melanogaster* to CAF, UMB, and TPH in exhibiting a robust ON response but no OFF response (Fig. 4B).

### Behavioral output correlates with molecular structure and OFF responses

**Fig. 7.**
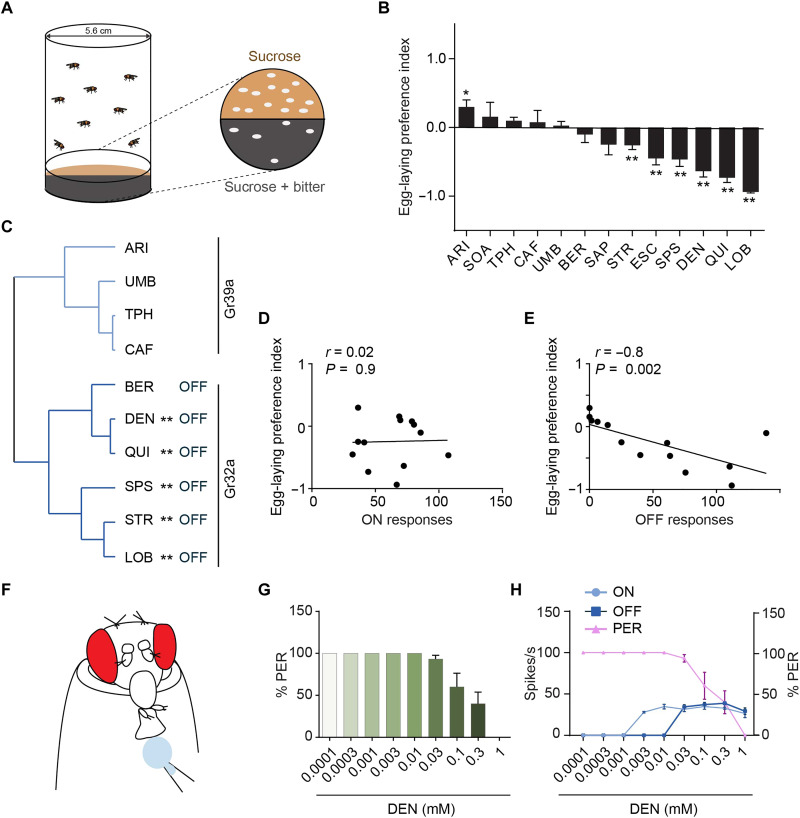
Behavioral output correlates with molecular structure and OFF responses. (**A**) The two-choice egg-laying assay. (**B**) Egg-laying preferences for 13 bitter compounds. Wilcoxon signed-rank test; *n* = 8 to 10. Error bars are SEM. The concentration of each of these compounds was 1 mM except that SAP was presented as 0.1%. *P < 0.05 and **P < 0.01. (**C**) Cluster analysis based on 32-physicochemical descriptors, Ward’s method. ** indicates an aversive tastant, as shown in (B). “OFF” indicates the tastant produced OFF responses, as shown in Fig. 5D. (**D**) Spearman correlation between egg-laying preference indices and ON responses. The ON response of each compound is the sum of the ON responses from I-a, S-a, and S-b. (**E**) Spearman correlation between egg-laying preference indices and OFF responses. The OFF response of each compound is the sum of the OFF responses from I-a, S-a, and S-b. (**F**) The labellar PER assay. A 100 mM sucrose stimulus is presented to the labellum; the percentage of stimulus presentations that produce a proboscis extension is indicated. (**G**) Labellar PER to 100 mM sucrose stimuli containing the indicated concentrations of DEN. *n* = 10. Error bars are SEM. (**H**) Comparison of dose dependence of PER, ON, and OFF responses. Data for ON and OFF responses are taken from Fig. 6A; PER responses are from (G).

The labellum plays a role in mediating the egg-laying preference for a number of taste compounds (*62*), and we therefore tested all 13 bitter compounds in a two-choice oviposition preference test. Female flies were given a choice between laying eggs on a substrate containing sucrose or a substrate containing sucrose and 1 mM of a bitter compound (Fig. 7A). From the number of eggs laid on each substrate, we computed a preference index. Six of the bitter compounds were aversive at this concentration: STR, ESC, SPS, DEN, QUI, and LOB (Fig. 7B).

We then classified the 13 tastants according to their structural similarity, using a set of molecular descriptors and a hierarchical cluster analysis (Fig. 7C and fig. S5). All five of the aversive tastants whose structure could be classified fell into the same cluster (the structure of ESC, SOA, and SAP could not be classified because the full set of their 32 physicochemical descriptors has not been determined). Thus, these aversive tastants were related in structure.

Notably, all six of the tastants in the larger cluster elicited OFF responses. None of the tastants in the smaller cluster elicited OFF responses. Thus, there is a correlation between OFF responses, behavior, and molecular structure.

In addition to these relationships, we note a final provocative observation. A recent study showed that UMB, TPH, and CAF, none of which elicited OFF responses, all depend on Gr39a, but not Gr32a, to elicit ON responses from I-b, S-a, and S-b bitter neurons (they elicit little if any response from I-a) (*6*). By contrast, DEN, QUI, SPS, STR, and LOB, all of which elicited OFF responses, all depend on Gr32a, but not Gr39a, to activate at least some GRNs (Fig. 7C) (*6*). This relationship suggests the possibility that Gr32a plays a role in OFF responses, perhaps providing a clue in future efforts to elucidate its mechanism.

We then asked whether the magnitude of the ON responses elicited by the 13 tastants at a 1 mM concentration correlated with their egg-laying preference indices, determined at the same concentration, and found no correlation (Fig. 7D; the mean responses of I-a, S-a, and S-b were summed). By contrast, the OFF responses of the 13 compounds correlated strongly with egg-laying aversion behavior (Fig. 7E; Spearman’s correlation, *R* = −0.8; *P* = 0.002).

We then examined another behavior that is driven directly by labellar neurons in a short time frame, i.e., seconds (*63*): the labellar proboscis extension response (PER) (Fig. 7F). In this paradigm, a sucrose solution is applied to the labellum, which drives an extension of the proboscis. The response is inhibited by the addition of bitter compounds to the sucrose solution. We examined the inhibition of the PER by DEN (Fig. 7G) across a broad concentration range and found that saturating levels of ON response were not sufficient to drive PER inhibition (Fig. 7H; the response of I-a neurons, which give the strongest response to DEN and are the most numerous class of bitter neurons, is plotted). PER inhibition was observed only at concentrations that elicited an OFF response (Fig. 7, G and H).

## DISCUSSION

We have elucidated a variety of noncanonical features of taste coding. Our results show that the taste system is much more sensitive than previously thought and has an unexpectedly rich capacity for encoding taste information.

### Spontaneous firing rates and dual response modes

Bitter, sugar, and water neurons all showed remarkably low levels of spontaneous firing—on the order of 1 or 2 spikes/s. The low firing frequencies may provide a means of increasing their sensitivity: A low concentration of tastant that elevates the firing frequency by a few spikes per second thereby produces a large fractional increase in activity.

The spontaneous firing rate of I-a neurons depends on Gr receptors it expresses. A simple model to explain this finding is that receptors exist in an equilibrium between an open state and a closed state and that the spontaneous firing frequency is governed by this equilibrium: Spontaneous firing is low if the fraction of receptors in the open state is low. When receptors are removed by mutation, the spontaneous rate may be reduced further. In a GRN such as I-a, with only a few Grs (*5*), deletion of individual Grs reduces the spontaneous rate appreciably; in GRNs with many receptors, such as S-a or S-b, removal of one receptor does not have a measurable effect on spontaneous firing.

We found that tastants can inhibit the spontaneous firing rate of GRNs. DEET particularly inhibited the spontaneous firing of the S-a neuron in mutant backgrounds. Thus, tastants can have two modes of action: activation and inhibition. One model to explain this inhibition is that binding of DEET to one or more receptors shifts the equilibrium from the open to the closed configuration. In summary, DEET is likely to bind to at least one receptor and activate it, explaining the increase in firing it elicits in wild type (Fig. 3B), but can also bind to another receptor and inactivate it, which is revealed in mutant backgrounds lacking a receptor that DEET activates. We note that tastants have previously been found to inhibit the excitatory responses of taste neurons to other tastants, but this is a distinct paradigm, and the mechanism of such inhibition may be different (*52*, *64*–*67*).

### Taste neurons as olfactory neurons

We found that taste neurons respond to the vapor of DEET, COU, and most members of a panel comprising 47 structurally diverse odorants. The magnitude of the responses depends on the neuron, the odorant, and the concentration; thus, the GRN repertoire provides a representation of the identity and intensity of the odorant. In summary, GRNs can function as ORNs.

The ecological significance of these olfactory responses is an interesting issue. The labellum is on the order of 200 to 240 μm in length in the antero-posterior direction, and sensilla range from ~15 to ~35 μm in length (*16*). We have found responses to DEET at distances of 1 to 50 μm (fig. S6), but, in nature, these responses to volatile compounds may be detected at greater distances depending on wind, temperature, and concentration. A taste sensillum of a fly that is exploring a potential food source in a microenvironment may often first encounter a compound via the air and shortly thereafter via contact. When contact is made, the stimulus may be part of a complex mixture that includes a wide variety of nonvolatile molecules, some of which could reduce the salience of the volatile cues. The effect of early airborne activation of taste neurons on decision-making awaits further study; in some cases, it could signal the presence of compounds at sufficiently high levels as to be aversive. In any case, our results suggest that Grs, in addition to binding a remarkable diversity of tastants, are also able to bind a wide range of odorants. Chemical compounds can evidently become solubilized in the lymph of taste sensilla and reach membrane receptors whether delivered to the sensillum via a solution or via the air.

### Sensitivity of the taste system

We have found that the taste system of *Drosophila* is much more sensitive to tastants in solution than previously thought (*2*, *5*, *6*, *14*, *17*–*19*, *21*, *23*–*25*, *29*, *48*, *49*, *52*, *53*, *56*, *59*). The sensitivity may be increased by the low spontaneous firing frequency, as noted above. High sensitivity may be especially adaptive by allowing detection of bitter compounds that are toxic at low levels. The taste system operates as an early warning system, protecting the fly from feeding or laying eggs on toxic food sources (*3*, *7*, *8*). A more sensitive system may provide more information about the suitability of a potential food source or oviposition substrate in nature.

Some sensory systems have evolved extraordinary sensitivity. Rod photoreceptors can signal the presence of a single photon (*68*). Moth pheromone-sensing neurons can detect a small number of pheromone molecules (*69*). In this regard, we note that tastant space is vast, and our results suggest the interesting possibility that other tastants of particular biological significance may be detected by the fly with even greater sensitivity than we have found here.

### OFF responses

We have documented electrophysiological OFF responses, at high cellular and temporal resolution, to a wide variety of tastants, in multiple taste neurons, and in six *Drosophila* species. OFF responses are elicited by a subset of bitter tastants in *D. melanogaster.* Among those tastants that elicit OFF responses, the identity of the tastant is encoded in the pattern of OFF responses it elicits from the different GRNs (Fig. 5D). The magnitude of the OFF response does not correlate with that of the corresponding ON response. The threshold for OFF responses is at least an order of magnitude higher than for ON responses for all bitter compounds tested.

We found surprising relationships between OFF responses, tastant structure, and behavior. Tastants that produced OFF responses in our study are structurally related to each other. The tastants that produced OFF responses have higher molecular weights than those that did not, with the exception of ARI. OFF responses correlated with behavior, in two different ways.

First, egg-laying preference could be predicted by the OFF response: Compounds that elicited larger OFF responses elicited greater aversion to egg laying (*R* = −0.8; *P* < 0.002). By contrast, there was no correlation between ON responses and egg-laying preference. These results suggest that the OFF response is a particularly salient feature of taste coding. We note that in establishing this correlation between OFF responses and egg-laying preference, we used the arithmetic sum of OFF (or ON) responses of I-a, S-a, and S-b bitter neurons as a simple representation of the response of the labellar GRN repertoire. In the future, it will interesting to expand this analysis to include other GRNs of the fly and to examine the correlations that emerge when their inputs are weighted in ways suggested by their connectivity in the central nervous system.

Second, PER behavior was inhibited only at concentrations when an OFF response was observed (Fig. 7H). This finding suggests a critical role for OFF responses in inhibiting the PER, a behavior that operates over a much shorter time scale than egg laying (*63*). In summary, our results from these two behavioral paradigms suggest that OFF responses provide an informative representation of both the identity and intensity of a tastant.

It is notable that we found a correlation between egg-laying avoidance behavior and OFF responses, but not ON responses. A priori one might have predicted that the onset of a bitter stimulus, producing an ON response, would elicit inhibition of egg laying, with a stronger ON response eliciting stronger inhibition. Rather, we have found that the offset of a bitter stimulus, producing an OFF response and signaling the appearance of a more suitable egg-laying site, may be a more salient signal. That is, to a fly exploring a site in nature, a large OFF response may reflect the end of a strong bitter stimulus and thereby indicate a large improvement in the suitability of the fly’s immediate locale for egg laying. One interpretation of our results is that egg-laying behavior is driven largely by a circuit that is activated by signals from the taste system indicating the appearance of a more favorable egg-laying site.

It is interesting that the same four *Gr* genes are required for both ON and OFF responses in I-a sensilla. One might have expected ON responses to be mediated by one complex of Grs and OFF responses by another. Rather, our results support a model in which a single Gr heteromultimeric complex has evolved an elegant mechanism of signaling both the onset and offset of taste stimuli. The evolution of dual function for a single complex is economical and may expand the coding capacity of a receptor repertoire of a given size.

It will be of great interest to elucidate the mechanism by which Grs produce ON and OFF responses. Determining the structure of a bitter Gr may be highly informative of mechanism, just as determination of Or structures has been (*70*, *71*). However, GRN signaling appears to have more degrees of freedom than ORN signaling. In addition to having OFF responses, which have not been reported in *Drosophila* ORNs, GRNs show different patterns of ON and OFF signaling within an individual neuron in response to different stimuli. For example, depending on the stimulus, an individual S-a neuron produces an ON but not an OFF response, an OFF but not an ON response, or both (Fig. 5D). This great complexity of signaling, which is unusual among sensory receptor neurons, may reflect the great complexity of receptor expression in GRNs (e.g., 29 Grs in S-a).

Together, the results of this study support the view that the taste system of *Drosophila* has a much greater coding capacity than previously thought. Taste neurons have greater dynamic ranges than previously appreciated, can be inhibited as well as activated, respond to many compounds before contact, and show OFF responses that link taste quality and quantity to behavioral output. The coding mechanisms described here will almost certainly be essential to an understanding of how chemical information is transformed by taste circuits into behavior.

### Limitations of the study

We have examined taste coding in a limited number of taste neurons, i.e., those of the labellum that are most accessible to electrophysiological analysis. Coding in other taste organs deserves much future exploration. Likewise, we have examined a diverse set of tastants and odorants, but chemical space is vast, and testing of other compounds may reveal additional features of taste coding. Our study examines monomolecular tastants, but, in nature, flies encounter complex mixtures of compounds. Much remains to be learned about the coding of taste mixtures.

## MATERIALS AND METHODS

### *Drosophila* stocks

Flies were reared on corn syrup and soy flour culture medium (Archon Scientific) at 25°C and 60% relative humidity in a 12:12-hour light-dark cycle. *Gr93a^3^* (LB27592), *Ir25a^2^* (LB41737), and *Ir76b^1^* (LB51309) were obtained from the Bloomington *Drosophila* Stock Center. *GR22f^1^* was obtained from S. J. Moon*. D. simulans* (14021-0251.001), *D. sechellia* (14021-0248.27), *D. erecta* (14021-0224.01), *D. mojavensis* (15081-1352.10), and *D. virilis* (15010-1051.00) were obtained from the *Drosophila* Species Stock Center. *Gr* deletions were generated as described in (*6*) and backcrossed to our control *w^1118^ Canton-S* line for five generations.

### Bitter tastants

Bitter tastants were obtained at the highest available purity from Sigma-Aldrich. All tastants were dissolved in water. For experiments comparing multiple genotypes, all fly lines were tested on the same day with an individual tastant. For electrophysiological recordings, tastants were tested at the following concentrations, unless otherwise indicated: 1 mM ARI, 1 mM berberine chloride (BER), 10 mM CAF, 1 mM DEN, 1 mM ESC, 1 mM (−)-lobeline hydrochloride (LOB), 1 mM QUI, 0.1% SAP from quillaja bark, 1 mM SOA, 10 mM strychnine nitrate salt (SPS), 1 mM sparteine sulfate salt (STR), 10 mM TPH, and 10 mM UMB. All compounds were stirred for 24 hours.

### Base recording

Flies were immobilized in pipette tips (200 μl), and the labellum was placed in a stable position on a glass coverslip. A reference tungsten electrode (catalog no. 716000, A-M Systems), electrolytically sharpened to 1 μm in tip diameter by dipping it repeatedly in a 10% KNO_3_ solution, was inserted into the eye of the fly. The recording tungsten electrode, identical to the reference electrode, was inserted gently into the base of a taste sensillum. Stimuli were delivered via a glass capillary (3 to 5 μm in tip diameter) to the taste hair using a motorized micromanipulator (EC1 60-0571 standard motorized control micromanipulator, Harvard Apparatus). Signals were amplified (10×; Syntech Universal AC/DC Probe; www.syntech.nl), sampled (10,667 samples/s), and filtered (100 to 3000 Hz with 50/60-Hz suppression) via a Universal Serial Bus-Intelligent Data Acquisition Controller (USB-IDAC) connection to a computer (Syntech). Action potentials were extracted using Syntech Auto Spike 32 software.

For the spontaneous firing activity experiments, the spontaneous activity was recorded for 100 s from each genotype. For the OFF response experiments, responses were quantified by counting the number of spikes generated over a 500-ms period after stimulus onset and offset. Response to the water diluent was not subtracted in any case; water elicited no response from bitter neurons as shown in Fig. 3B. When recording from sensilla of a particular class, e.g., I-a, all sensilla of that class, i.e., I0 to I6, were tested. Five- to 7-day-old mated female flies were used.

The response to the vapors of chemical compounds was calculated as a peak response, which refers to the maximum spike frequency reached in 250-ms bins. The distance from the tip of the stimulus capillary to the tip of a taste sensillum was 1 μm. DEET also elicited responses at distances of 10, 25, and 50 μm from the sensillum tip, but not at a distance of 100 μm (fig. S6).

### Two-choice oviposition assay

The two-choice oviposition assay was modified from (*72*), except that corn meal food was replaced with 1% agar containing 100 mM sucrose. Oviposition plates consisted of plastic petri dishes (60 × 15 mm; Falcon), which were divided into two halves; each half contained either sugar or sugar mixed with a bitter compound. Twenty-five newly eclosed flies (5 males and 20 females) were transferred to fly food vials and kept at 25°C and 60% relative humidity in a 12:12-hour light-dark cycle. Flies, when 5 to 7 days old, were placed into an oviposition cage (Genesee Scientific) without anesthesia through a small funnel that fits in the lid of the cage and left for 24 hours in the dark. Eggs were counted from each substrate. An oviposition preference index was calculated as follows: (number of eggs on sucrose with bitter substrate − number of eggs on sucrose alone substrate)/(total number of eggs on both substrates).

### PER assay

PER assays were carried out as described in Slone *et al.* (*73*) and Ahn *et al.* (*74*) with some modifications. Briefly, flies were collected on the day of eclosion and kept on the corn syrup and soy flour culture medium for 3 to 5 days at 25°C. Before performing PER assays, mated female flies were starved for 24 hours at 25°C in vials with water-saturated kimwipes. Flies were then mounted inside pipette tips and allowed to recover for 30 min at room temperature. Before the PER assay, flies were allowed to drink water until satiation to ensure that PER responses were derived from nutrients. Taste solutions were delivered with a 10-ml pipette to the labellum for up to ~4 s. Each fly was tested three times with one individual taste solution, and flies were allowed to drink water between each new application. A PER was recorded as positive (*1*) if the proboscis was fully extended, otherwise it was recorded as negative (0). PER response scores (%) from a single fly were 0% (zero of three responses in the three applications), 33% (one of three), 66% (two of three), or 100% (three of three). Each concentration of DEN was prepared in 100 mM sucrose solution.

### Quantification and statistical analysis

Hierarchical cluster analyses were performed using Ward’s method with PAST [paleontological statistics software package for education and data analysis; Hammer *et al.* (*75*)]. Other statistical tests were performed in GraphPad Prism (version 6.01). All error bars are SEM. Molecular descriptors were calculated by Dragon (www.talete.mi.it). Descriptors were *z* scores normalized for principal components analysis.
