# Supplementary material for: Diverse mechanisms of taste coding in Drosophila
Source: Sci Adv. 2023 Nov 17;9(46):eadj7032. doi: 10.1126/sciadv.adj7032 (PMC10656072; doi:10.1126/sciadv.adj7032)
Supplement: Supplementary file 1 — Figs. S1 to S6 [file sciadv.adj7032_sm.pdf]

Science Advances
AAAS

Supplementary Materials for

Diverse mechanisms of taste coding in *Drosophila*

Hany K. M. Dweck and John R. Carlson

Corresponding author: John R. Carlson, john.carlson@yale.edu;
Hany K. M. Dweck, hany.dweck@yale.edu, hany.dweck@ct.gov

Sci. Adv. **9**, eadj7032 (2023)
DOI: 10.1126/sciadv.adj7032

This PDF file includes:

Figs. S1 to S6

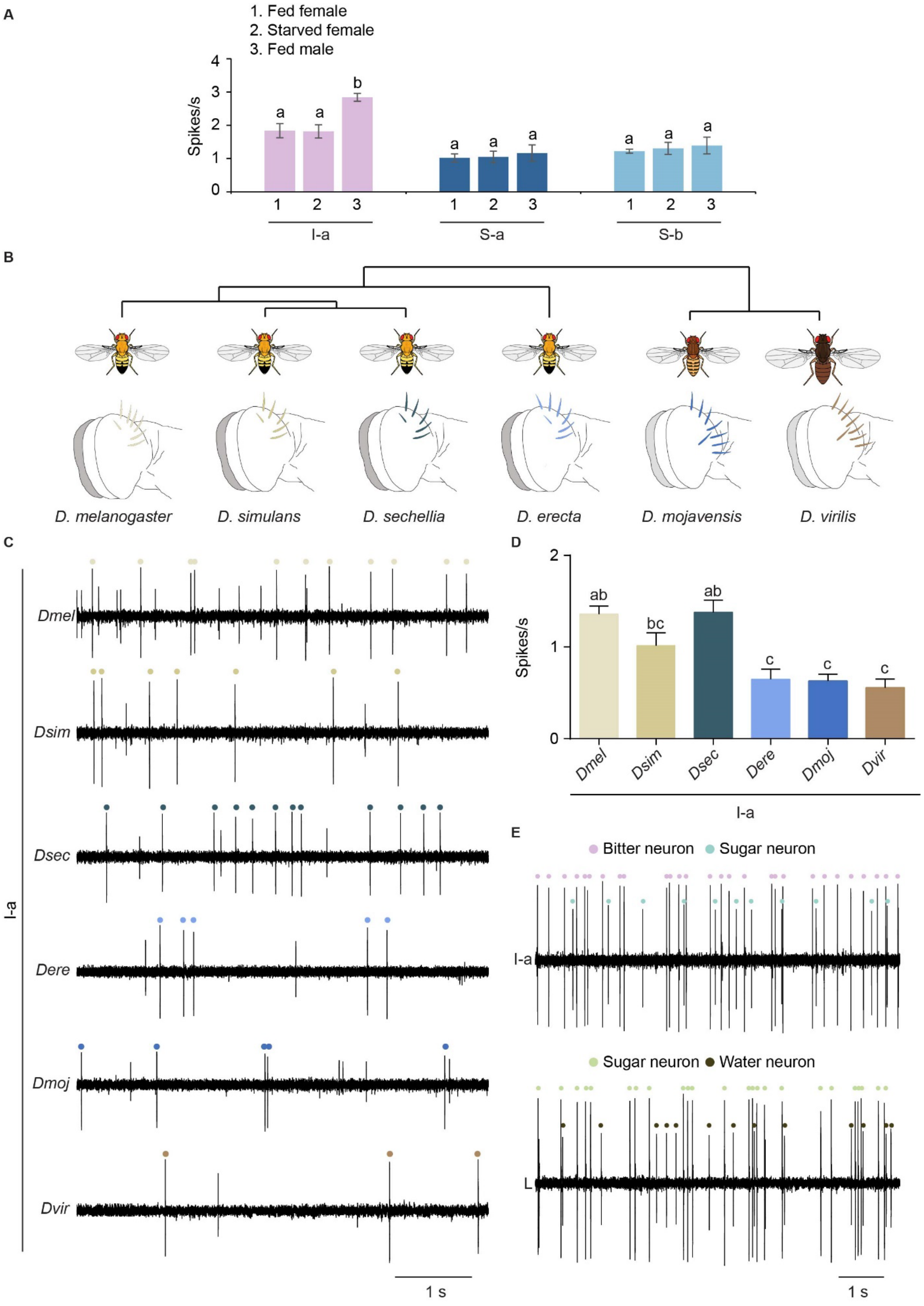
A
1. Fed female
2. Starved female
3. Fed male
Spikes/s
I-a
S-a
S-b
B
D. melanogaster
D. simulans
D. sechellia
D. erecta
D. mojavensis
D. virilis
C
Dmel
Dsim
Dsec
Dere
Dmoj
Dvir
I-a
1 s
D
Spikes/s
Dmel
Dsim
Dsec
Dere
Dmoj
Dvir
I-a
E
Bitter neuron
Sugar neuron
I-a
Sugar neuron
Water neuron
L
1 s

Figure S1. Low spontaneous firing rates of I-a bitter neurons are a conserved feature among drosophilid species. Related to Figure 2.
(A) Spontaneous activities of I-a, S-a, and S-b bitter neurons in fed female, starved female, and fed male *D. melanogaster.* One-way ANOVA followed by Dunnett's multiple comparison test; n = 5-7. Bars with "a" and "b" above are significantly different. Error bars are SEM.

(B) Position of I-a sensilla on labella of six drosophilid species.

(C) Example traces of spontaneous activities in *Dmel*, *Dsim*, *Dsec*, *Dere*, *Dmoj*, and *Dvir*.

(D) I-a spontaneous activities of six drosophilid species. One-way ANOVA followed by Tukey's multiple comparison test; n = 5-12. Bars with a common letter are not distinguishable. Error bars are SEM.

(E) Outstanding example traces of I-a (top), showing bitter (large amplitude) and sugar (small amplitude) neurons, and of L sensilla (bottom), showing sugar (large amplitude) and water (small amplitude) neurons. We note that the trace from the L sensillum is atypical; in most traces the two spike amplitudes are more difficult to resolve.

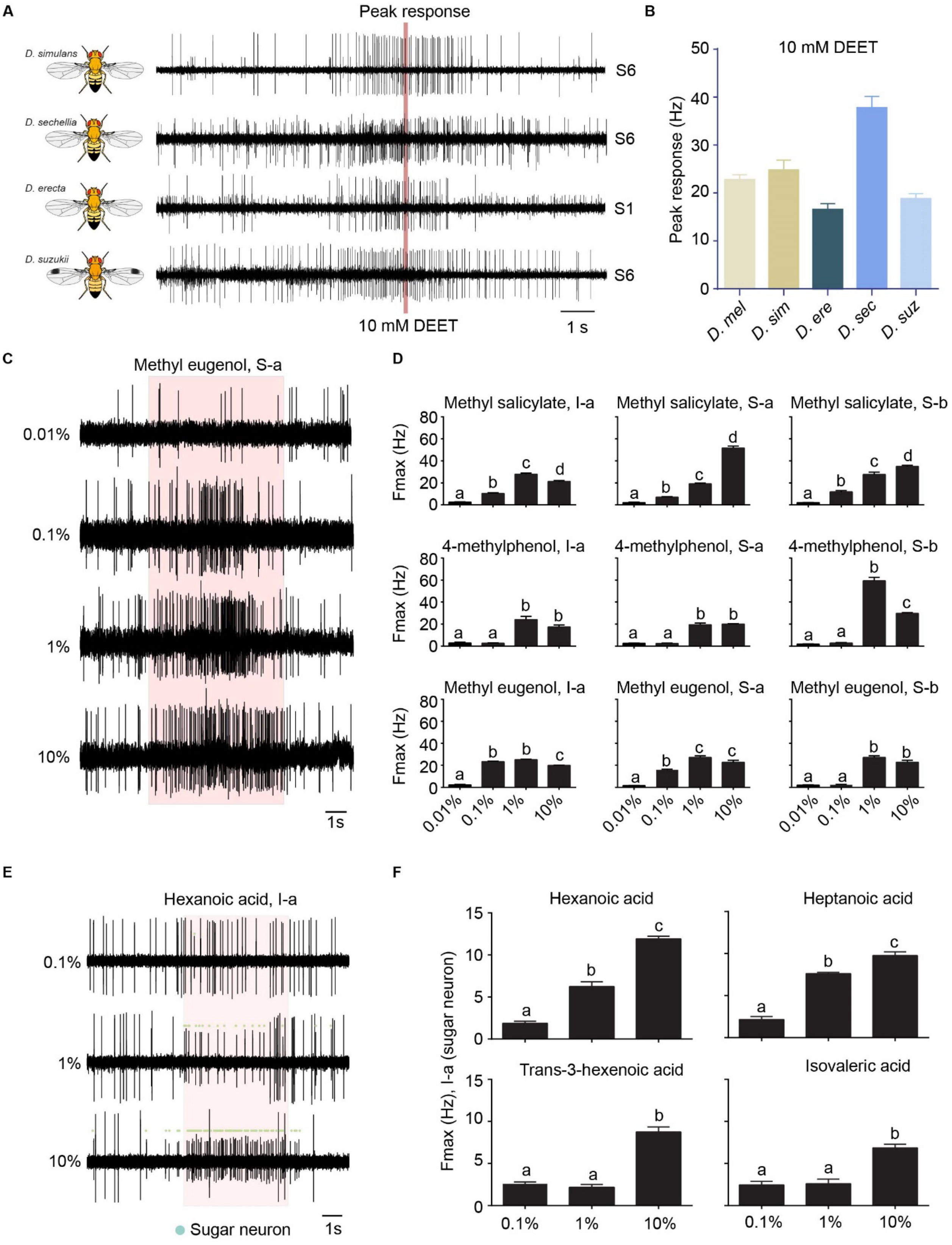

A
Peak response
D. simulans
D. sechellia
D. erecta
D. suzukii
S6
S6
S1
S6
10 mM DEET
1 s
B
10 mM DEET
Peak response (Hz)
D. mel
D. sim
D. ere
D. sec
D. suz
C
Methyl eugenol, S-a
0.01%
0.1%
1%
10%
1s
D
Methyl salicylate, I-a
Methyl salicylate, S-a
Methyl salicylate, S-b
4-methylphenol, I-a
4-methylphenol, S-a
4-methylphenol, S-b
Methyl eugenol, I-a
Methyl eugenol, S-a
Methyl eugenol, S-b
Fmax (Hz)
E
Hexanoic acid, I-a
0.1%
1%
10%
Sugar neuron
1s
F
Hexanoic acid
Heptanoic acid
Trans-3-hexenoic acid
Isovaleric acid
Fmax (Hz), I-a (sugar neuron)

Figure S2. Responses to the vapors of volatile compounds. Related to Figure 3.
(A) Example traces of S-a in *D. stimulus, D. sechellia, D. erecta,* and *D. suzukii* in response to the vapor of 10 mM DEET.

(B) Responses of S-a in *D. melanogaster, D. stimulus, D. sechellia, D. erecta,* and *D. suzukii* to the vapor of 10 mM DEET. n = 5-10. Error bars are SEM.

(C) Example traces of S-a in response to the vapor of different percent concentrations of methyl eugenol.

(D) Responses of I-a, S-a, and S-b to the vapor of different percent concentrations of methyl salicylate, 4-methylphenol, and methyl eugenol. One-way ANOVA followed by Tukey's multiple comparison test; n = 5. Bars with the same letter are not distinguishable. Error bars are SEM.

(E) Example traces of I-a in response to the vapor of different percent concentrations of hexanoic acid. The sensillum contains two neurons; the one that responds is the one with the smaller spike.

(F) Responses of I-a to the vapor of different percent concentrations hexanoic acid, heptanoic acid, trans-3-hexenoic acid, and isovaleric acid. One-way ANOVA followed by Tukey's multiple comparison test; n = 5. Significant differences are denoted by letters. Error bars are SEM.

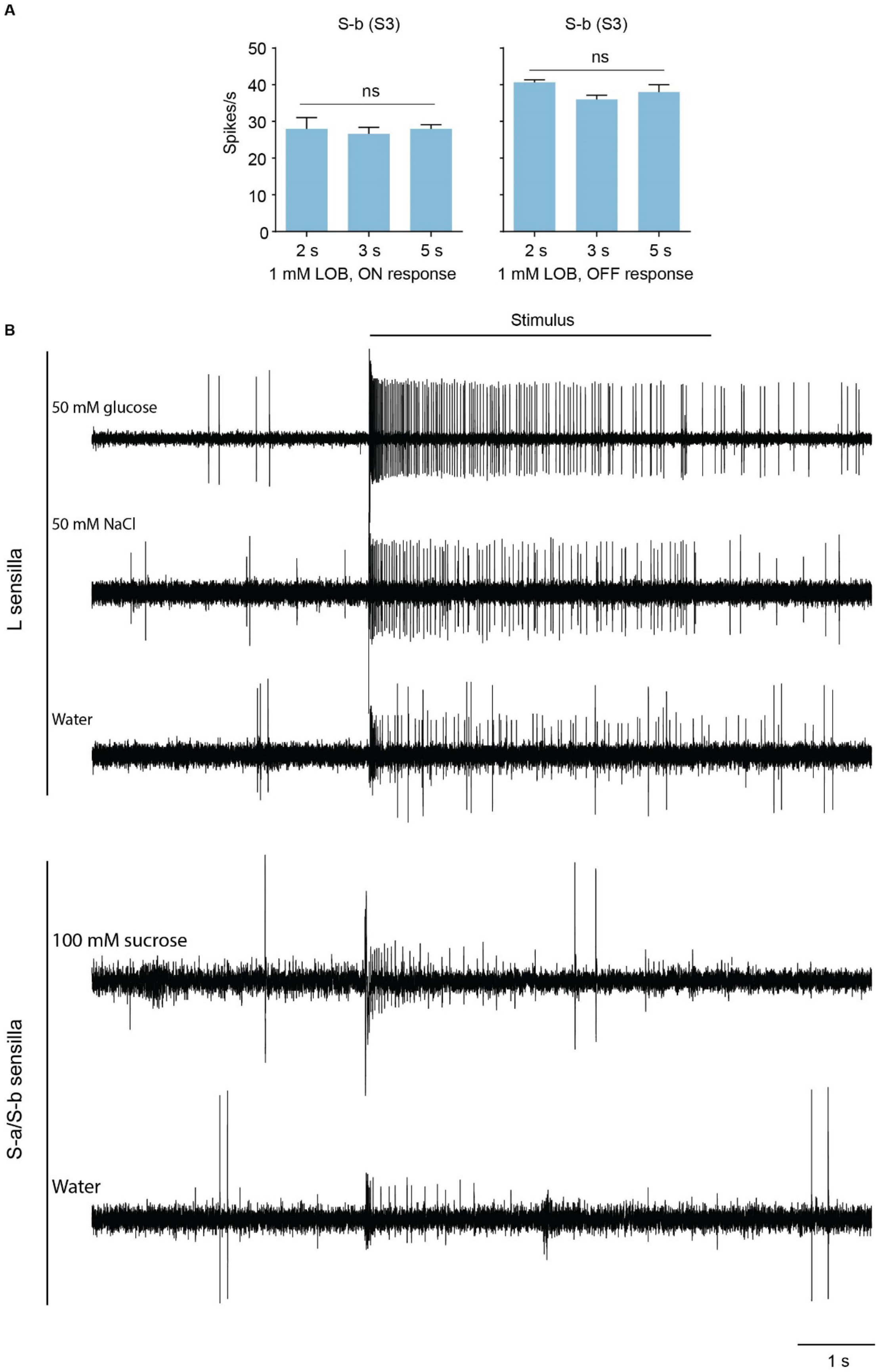
A
S-b (S3)
S-b (S3)
Spikes/s
50
40
30
20
10
0
ns
ns
2 s
3 s
5 s
1 mM LOB, ON response
2 s
3 s
5 s
1 mM LOB, OFF response
B
Stimulus
L sensilla
50 mM glucose
50 mM NaCl
Water
S-a/S-b sensilla
100 mM sucrose
Water
1 s

Figure S3. Durations of responses to LOB and responses to glucose, NaCl, sucrose, and water. Related to Figure 5.
(A) ON and OFF responses to LOB with different stimulus durations. $p > 0.05$; One-way ANOVA followed by Tukey's multiple comparison test; $n = 5$. Error bars are SEM.

(B) The traces, all from *D. melanogaster,* show responses of the indicated sensilla to the indicated tastants. Note that the amplitudes of the water-sensitive and sucrose-sensitive neurons are smaller than those of bitter- or NaCl-sensitive neurons. In no case do we observe an OFF response.

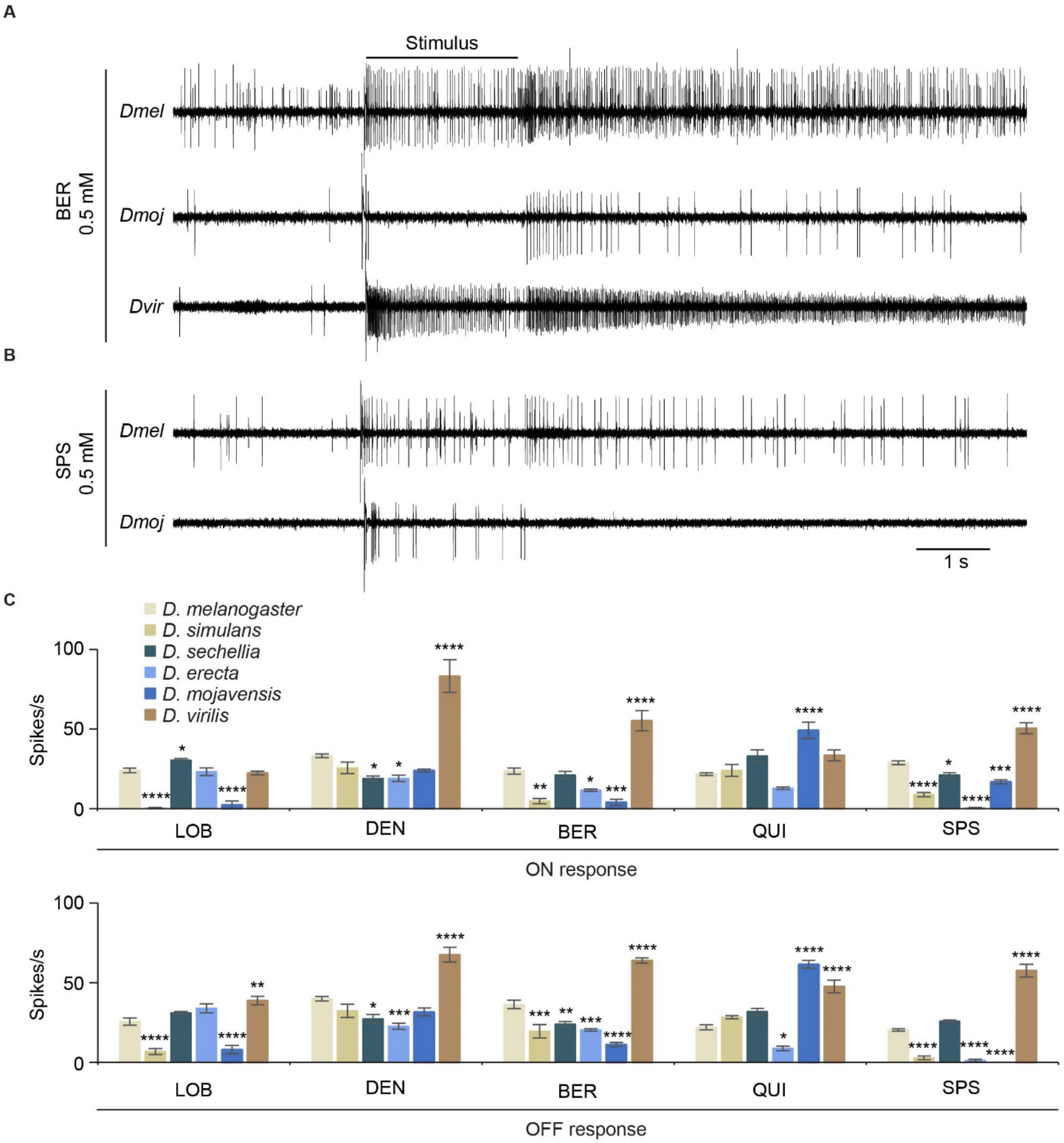
A
Stimulus
BER 0.5 mM
Dmel
Dmoj
Dvir
B
SPS 0.5 mM
Dmel
Dmoj
1 s
C
D. melanogaster
D. simulans
D. sechellia
D. erecta
D. mojavensis
D. virilis
Spikes/s
100
50
0
LOB
DEN
BER
QUI
SPS
ON response
OFF response

Figure S4. Evolutionary shifts in OFF responses. Related to Figure 6.
(A) Example traces of the response of I-a in *D. melanogaster*, *D. mojavensis*, and *D. virilis* to 0.5 mM BER.

(B) Example traces of the response of I-a in *D. melanogaster* and *D. mojavensis* to 0.5 mM SPS.

(C) I-a ON and OFF responses to DEN, BER, LOB, QUI, and SPS in six drosophilid species, highlighting differences in responses of other species to those of *D. melanogaster.* *p < 0.05; **p < 0.01; ***p < 0.001; ****p < 0.0001; one-way ANOVA followed by Dunnett's multiple comparison test; n = 5-7. Error bars are SEM.

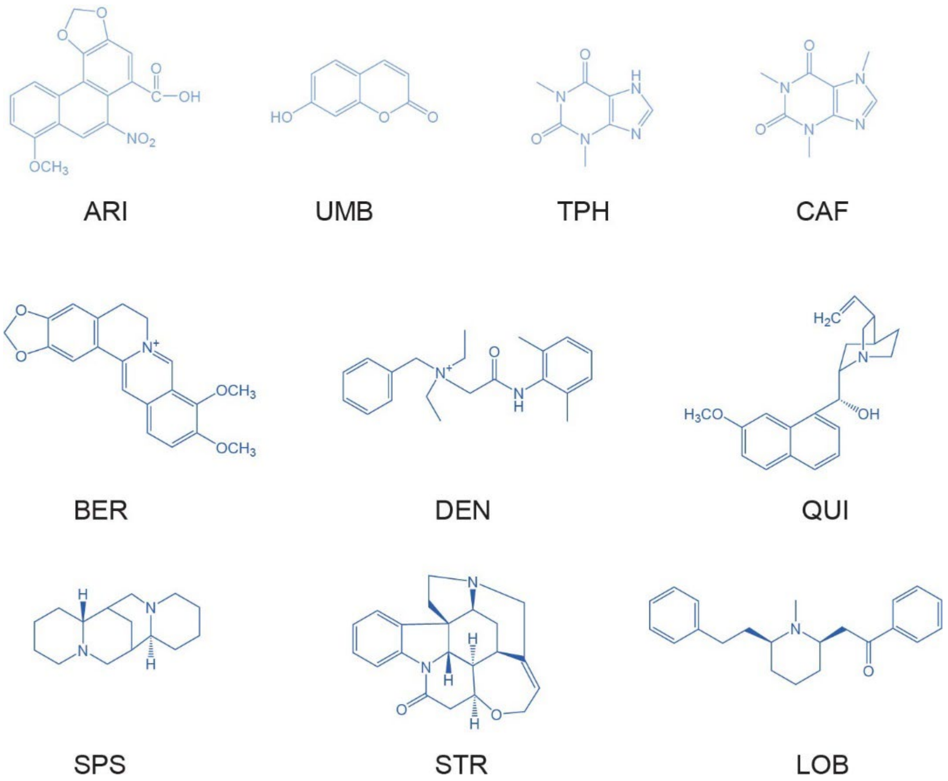

Figure S5.
Structures of tastants analyzed in Fig. 7C.

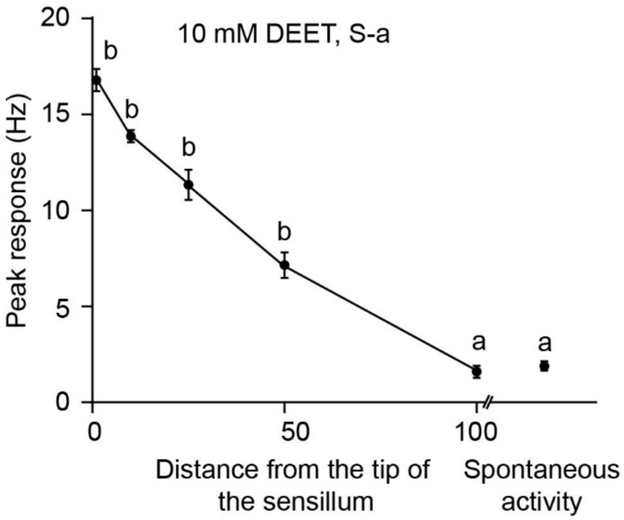

Figure S6. Vapor of 10 mM DEET activates the bitter neuron at different distances from the tip of an S-a sensillum. Spontaneous firing rate of bitter neurons of S-a is shown at right. One-way ANOVA followed by Dunnett’s multiple comparison test; n = 5. Mean values indicated with "a" and "b" are significantly different. Error bars are SEM.
